# Outlier-tolerant relative positioning method based on multi-source information fusion for unmanned aerial vehicles

**DOI:** 10.1038/s41598-025-00923-5

**Published:** 2025-05-10

**Authors:** He Song, Yang Bi, Shaolin Hu, Yilei Chen

**Affiliations:** 1 School of Electronic Engineering, Xihang University, Xi’an, 710077 China; 2 the Key Laboratory of Intelligent Perception and Processing Technology for Aircraft Multimodal Heterogeneous Information in Higher Education Institutions of Shaanxi Province, Xi’an, 710077 China; 3 School of Automation, Guangdong University of Petrochemical Technology, Maoming, 525000 China

**Keywords:** Electrical and electronic engineering, Computer science

## Abstract

Relative positioning is a key technology that needs to be addressed for unmanned aerial vehicles (UAVs) to achieve flight mission involving autonomous aerial refueling, cluster formation and cooperative control. To address the shortcomings of the least squares (LS)-based multi-source information fusion method, such as poor outlier-tolerance, the idea of outlier-tolerance is used to improve the LS method. A novel loss function is proposed by replacing the parabolic function with a piecewise function, and a multi-source information outlier-tolerant relative positioning method based on the novel loss function is established. The simulation results show that the established method has a good outlier tolerance ability, which can avoid the adverse effects of outliers and ensure the reliability of the calculation results without significantly affecting the accuracy of the relative positioning.

## Introduction

Relative positioning is a key technology for UAVs to achieve cooperative formation and autonomous aerial refueling^1,2^. If the relative location information between UAVs cannot be obtained accurately or if the accuracy of relative positioning obtained is low, it will seriously affect the execution efficiency of flight tasks^3,4^. Therefore, a high-precision relative positioning method is highly important for ensuring the successful completion of a mission.

Initially, researchers proposed numerous relative positioning methods between tankers and receivers on the basis of a single measurement source. However, with the demonstration of experimental applications, researchers quickly realized the shortcomings and deficiencies of using a single measurement source for relative positioning^5^. For example, because external electromagnetic interference can affect the quality of radar measurement data, relative positioning methods based on radars can reduce relative positioning accuracy^6,7^. The relative positioning method based on the BeiDou Navigation Satellite System (BDS) or Global Positioning System (GPS)^8,9^ may have significant drawbacks such as unstable received signals and susceptibility to external interference, which can affect the accuracy of relative positioning^10^. Owing to the cumulative bias of measuring devices such as gyroscopes, accelerometers, and magnetometers, the relative positioning method, which is based on inertial navigation systems (INSs) or inertial measurement units (IMUs)^11,12^, can result in significant relative positioning errors and reduce the relative positioning accuracy. Although the relative positioning method of Vision Navigation (VisNav), which is based on feature light points or image matching^13,14^, does not rely on satellite navigation signals, it has problems such as time delay and susceptibility to external flight environments and limitations due to the need to process large amounts of data, which can reduce the relative positioning accuracy and even make the method unsuitable for ultralong-distance situations^15^. With the development of aerial refueling technology and the expansion of application fields, increasingly high requirements for the accuracy, real-time reliability, and autonomous intelligence of the relative positioning method of tankers/receivers have been proposed. The traditional relative positioning method between the tanker and receiver, which is based on a single measurement source device, is no longer sufficient to meet these high-performance requirements.

To further improve the accuracy of relative positioning, researchers have proposed a relative positioning method based on multi-source information fusion for tankers/receivers. The multi-source information fusion method can obtain the optimal estimation results by making full use of the information of measured equipment to learn from each other’s strengths and weaknesses, and it can not only improve the accuracy of estimation results, but also improve the reliability of systems^16^. Therefore, the multi-source information fusion method has been widely applied in high-precision relative positioning technology for UAVs and has achieved a series of research results^17–24^. For example, Wang designed a distributed filtering information fusion architecture using a VisNav/INS/DGPS combined relative positioning scheme, which improved the relative positioning accuracy of the tanker/receiver^25^. Wilson combined infrared cameras and active infrared light-emitting diode markers as auxiliary relative positioning devices based on GPS/INS, achieving high-precision relative positioning for tanker/receiver formation flight, and experimental results revealed that the root mean square errors of relative positioning in the horizontal and vertical directions were reduced to 1.2 m and 0.44 m, respectively^26^. Gross used Ultra-Wide Band (UWB) for assisted relative positioning based on DGPS/INS, the UWB relative measurement information was used to correct the integer ambiguity of the carrier phase, and simulation results showed that the proposed method not only improved the relative positioning accuracy, but also enhanced the stability of system motion^27^. Wang combined the advantages of the GPS and BDS to design a relative positioning scheme based on the GPS/BDS, which can achieve high-precision relative positioning of the tanker/receiver^28^. Fu used a tight combination of the BDS and INS for the relative positioning of tankers/receivers, the measurement information was fused through an extended Kalman filter and the experiment proved that the proposed method can achieve relative positioning accuracy at the decimeter level and avoid the adverse effects of signal interruption on relative positioning, ensuring the reliability of the output results^29^.

The LS method is a common method for multi-source information fusion that estimates the optimal state of the system by processing measurement data centrally^30^. Owing to the effect and influence of various accidental factors, abnormal data, such as outliers or spot data, often exist in a measurement dataset that deviates substantially from the true value of the target^31^. Without distinguishing the quality of measurement data, the LS-based multi-source data fusion method directly processes measurement data containing abnormal data in a centralized manner, resulting in significantly greater adverse effects than normal data points during the calculation process, and seriously affecting the accuracy and reliability of the calculation results. To address this issue, the idea of outlier-tolerant is used to improve the loss function of the traditional LS, an outlier-tolerance LS algorithm with outlier-tolerance ability is designed and an outlier-tolerant relative positioning method based on multi-source information fusion for UAVs is established. The established method can effectively avoid the adverse effects caused by abnormal measurement data, ensure the reliability of relative positioning results, and improve the outlier-tolerance ability.

The structure of this paper is as follows. First, the outlier-tolerant LS method is designed. Second, the Beidou navigation system (BDS) receiver, the global positioning system (GPS) receiver and the vision navigation system (VisNav) are selected as the measurement sources of the relative positioning for UAVs, and an outlier-tolerant relative positioning method based on multi-source information fusion is established. Third, the performance of the proposed method with numerical simulation and result analysis is verified. Finally, some conclusions of this research are given.

## Outlier-tolerant improvement of the LS method

Assuming that the theoretical value of a motion body is *Ey* and that the corresponding measured data are *y*, the LS method essentially uses the quadratic loss function shown in formula (1) to centrally process a series of measured data according to formula (2) to obtain the optimal estimate $\hat {y}$ of the theoretical value^32^.

1 $$f\left( e \right)=\frac{1}{2}{e^2}=\frac{1}{2}{\left( {y - Ey} \right)^2}$$

2 $$\hat {y}=\arg \mathop {\hbox{min} }\limits_{{Ey}} \sum\limits_{y} {{{\left( {y - Ey} \right)}^2}}$$

where, *e* is the error.

### Analysis of the impact of abnormal data on LS

The measurement dataset of equipment inevitably contains random errors and a small portion of abnormal data during the measurement process. The manifestation of abnormal data is that the absolute value of the difference between the measured value and the true value is significantly greater than the absolute value of the random error, often referred to as outliers, and outliers that appear in series are referred to as spots in this article.

For both outliers and spots, the contribution of formula (2) is significantly greater than the impact of normal data. The reason is that formula (2) is a quadratic loss function, and the greater the deviation between the measured data and the true value is, the greater the impact. The actual result is that the influence of outliers will cause distortion in the estimated results. Thus, the traditional LS method lacks outlier tolerance for abnormal data. To control the adverse effects of abnormal data, it is necessary to limit the output size of formula (1).

### Design of the outlier-tolerant LS method

To overcome the limitations of the traditional LS method, in this section, an outlier-tolerant LS method with outlier tolerance ability is constructed by changing the loss function. Specifically, drawing on the calculation ideas of the existing fitting smooth differential method^33^ and on the engineering definition of measurement outliers (3–5*σ*, where *σ* is the standard deviation of the measurement data), the specific design process of the outlier-tolerant LS method is as follows.

(1) When $\left\| e \right\| \leqslant 3\left\| \sigma \right\|$, the measured data are considered normal. At this time, the loss function still uses the original loss function:

3 $${f_p}=\frac{1}{2}{\left\| e \right\|^2}$$

(2) When $3\left\| \sigma \right\|<\left\| e \right\| \leqslant 4\left\| \sigma \right\|$, the measured data are considered abnormal. In this case, the loss function is corrected to:

4 $${f_p}=a \cdot \left\| e \right\| \cdot \left\| \sigma \right\|$$

According to ${f_p}=\frac{1}{2} \cdot 3\left\| \sigma \right\| \cdot 3\left\| \sigma \right\|=4.5{\left\| \sigma \right\|^2}$, we can obtain *a* = 1.5.

(3) When $4\left\| \sigma \right\|<\left\| e \right\| \leqslant 5\left\| \sigma \right\|$, there are large outliers in the measurement data. To reduce the influence of measurement outliers, the loss function is modified as:

5 $${f_p}= - {\left( {\left\| e \right\| - 5\left\| \sigma \right\|} \right)^2}+b{\left\| \sigma \right\|^2}$$

According to ${f_p}=1.5 \cdot 4\left\| \sigma \right\| \cdot \left\| \sigma \right\|=6{\left\| \sigma \right\|^2}$, we can obtain *b* = 7, and substituting *b* = 7 into formula (5) results in ${f_p}= - {\left\| e \right\|^2}+10\left\| e \right\|\left\| \sigma \right\| - 18{\left\| \sigma \right\|^2}$.

(4) When $\left\| e \right\|>5\left\| \sigma \right\|$, there are outliers in the measured data that deviate significantly from the true data. To ensure the accuracy of the calculation results, at this point, the loss function is corrected to a constant value, i.e.,

6 $${f_p}=c{\left\| \sigma \right\|^2}$$

According to${f_p}= - {\left( {5\left\| \sigma \right\| - 5\left\| \sigma \right\|} \right)^2}+7{\left\| \sigma \right\|^2}=7{\left\| \sigma \right\|^2}$, we can obtain *c* = 7.

On the basis of the above analysis and calculation, the novel loss function of the outlier-tolerant LS method is shown in formula (7).

7 $${f_p}=\left\{ {\begin{array}{*{20}{c}} \begin{gathered} \frac{1}{2}{\left\| e \right\|^2}, \hfill \\ 1.5\left\| e \right\|\left\| \sigma \right\|, \hfill \\ - {\left\| e \right\|^2}+10\left\| e \right\|\left\| \sigma \right\| - 18{\left\| \sigma \right\|^2}, \hfill \\ 7{\left\| \sigma \right\|^2}, \hfill \\ \end{gathered} &\begin{gathered} \left\| e \right\| \leqslant 3\left\| \sigma \right\| \\ \\ 3\left\| \sigma \right\|<\left\| e \right\| \leqslant 4\left\| \sigma \right\| \\ 4\left\| \sigma \right\|<\left\| e \right\| \leqslant 5\left\| \sigma \right\| \\ \left\| e \right\|>5\left\| \sigma \right\| \\ \end{gathered} \end{array}} \right.$$

The improved loss function in formula (7) allows for a reasonable quantitative transition from normal data to abnormal data during the calculation process; it does not need to eliminate abnormal data, so that it can ensure that the measured data are evenly spaced. Moreover, the effective information contained in it is fully utilized to improve the outlier tolerance ability of the method and ensure the reliability of the calculation results.

### Performance analysis of the outlier-tolerant LS method

**Fig. 1:**
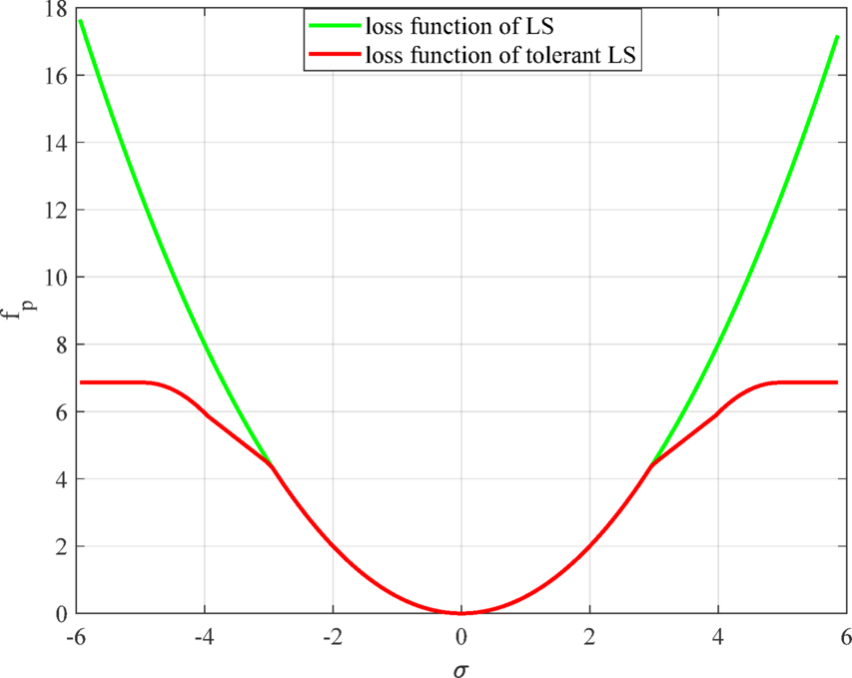
Comparison of loss functions.

By modifying the loss function of the traditional LS method through formula (7), the measurement data can be treated more reasonably and fairly. Using the error *e* as the independent variable and the loss function value *f* as the output variable, the standard deviation of the measurement data with *σ* = 0.99 is selected and plotted according to Eqs. (1) and (7). A comparison of the results is shown in Fig. 1.

Figure 1 clearly shows that the improved loss function can effectively overcome the lack of outlier tolerance ability of the traditional quadratic loss function for abnormal measurement data, and can distinguish and utilize measurement data, indicating that the method has good outlier tolerance ability.

Through comparative analysis, when formula (7) is used instead of the loss function of the traditional LS method for calculation, it has the following advantages:

(1) When the measured data are normal $(\left\| e \right\| \leqslant 3\left\| \sigma \right\|)$, and the loss function is $f\left( e \right)=\frac{1}{2}{\left\| e \right\|^2}$, the calculation results of the outlier-tolerant LS method are similar to those of the traditional LS method, ensuring the high accuracy of the calculation results when the data are normal.

(2) When the measured data are abnormal $(\left\| e \right\|>5\left\| \sigma \right\|)$, the loss function is $f\left( e \right)=7{\left\| \sigma \right\|^2}$, and it is a constant and can avoid the adverse effects of abnormal data and ensure the reliability of the calculation results.

## Multi-source information fusion-based outlier-tolerant relative positioning method

In this section, the outlier-tolerant LS method is applied to the relative positioning of two UAVs via multi-source information fusion, to avoid the adverse impact of abnormal measurement data on the accuracy of relative positioning and ensure the reliability of the relative positioning results.

### Establishment of relative position model

The measurement sources for the relative positioning of UAVs in this paper mainly include BDS receivers, GPS receivers, and VisNav devices. To study the relative positioning method based on multi-source information fusion, we must evaluate the relative measurement principles of different measurement sources and establish the corresponding measurement models.

In the process of building relative measurement models, conversion between different coordinate systems is required to achieve unification of coordinate systems. Considering what is studied in this paper, the coordinate systems involved mainly include the following:

Geodetic coordinate system *I* (*O*-*XYZ*): the origin of the coordinate system *O* is the center of the reference ellipsoid, the *Z*-axis points to the north pole of the reference ellipsoid, the *X*-axis points to the intersection of the starting meridian plane and the equator, the *Y*-axis is perpendicular to the *XOZ* plane, and the *Z*-axis and *X*-axis form a right-handed coordinate system.

Body coordinate system *A* (*O*_*A*_-*X*_*A*_*Y*_*A*_*Z*_*A*_): The origin of the coordinate system *O*_*A*_ is the center of mass of the body, the *Z*-axis points to the top of the body, the *X*-axis points in the direction of the body forward, the *Y*-axis points to the left side of the body, and the *Z*-axis and *X*-axis form a right-handed coordinate system.

Body coordinate system *B* (*O*_*B*_-*X*_*B*_*Y*_*B*_*Z*_*B*_): the origin of the coordinate system *O*_*B*_ is the center of mass of the body, the *Z* axis points to the top of the body, the *X* axis points in the direction of the body forward, the *Y* axis points to the left side of the body, and the *Z* axis and *X* axis form a right-handed coordinate system.

(1) BDS measurement model.

**Fig. 2:**
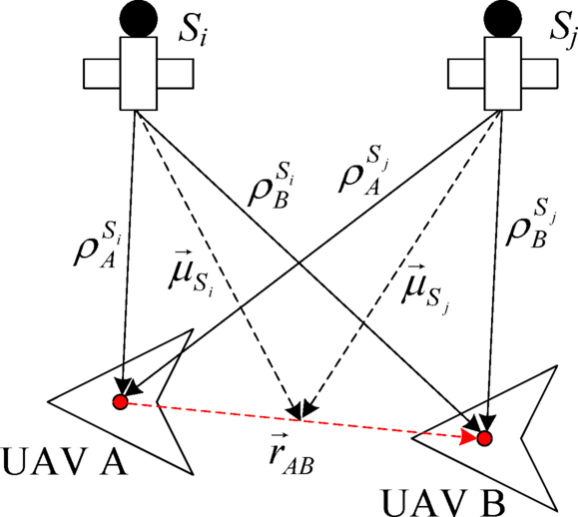
Measurement principal of the relative differential pseudorange.

Assuming that both UAVs A and B are equipped with a BDS receiver installed at the center of mass of the aircraft, the BDS receiver is located at the origin of body coordinate systems A and B, and can be viewed as a particle with the UAV in the geodetic coordinate system. The measurement output of the BDS receiver is the pseudorange $\rho$ between the receiver and the satellite relative to the geodetic coordinate system. In this work, we use the pseudorange relative difference method to establish a measurement model between the BDS-measured output and the relative location, the measurement principle is shown in Fig. 2.

Specifically, the pseudoranges $\rho _{A}^{{{S_i}}}$ and $\rho _{B}^{{{S_i}}}$ between the UAV A and B airborne BeiDou receivers and the satellite *S*_*i*_(*i* = 1,2,……,*N*) are defined as^34^:

8 $$\left\{ \begin{gathered} \rho _{A}^{{{S_i}}}=r_{A}^{{{S_i}}}+c\left( {\delta {t_A} - \delta {t_{{S_i}}}} \right)+\varepsilon _{A}^{{{S_i}}}+\varepsilon _{{A1}}^{{{S_i}}}+\varepsilon _{{A2}}^{{{S_i}}}+\varepsilon _{{A3}}^{{{S_i}}} \hfill \\ \rho _{B}^{{{S_i}}}=r_{B}^{{{S_i}}}+c\left( {\delta {t_B} - \delta {t_{{S_i}}}} \right)+\varepsilon _{B}^{{{S_i}}}+\varepsilon _{{B1}}^{{{S_i}}}+\varepsilon _{{B2}}^{{{S_i}}}+\varepsilon _{{B3}}^{{{S_i}}} \hfill \\ \end{gathered} \right.$$

where, $r_{A}^{{{S_i}}}$ and $r_{B}^{{{S_i}}}$ are the geometric distances between UAVs A and B to satellite *S*_*i*_; $\delta {t_A}$ and $\delta {t_B}$ are the time differences between the airborne BDS receivers on UAVs A and B; *c* and $\delta {t_{{S_i}}}$ are the variances between the vacuum velocity and satellite clock time difference; $\varepsilon _{A}^{{{S_i}}}$ is the airborne BDS receiver used to measure noise; $\varepsilon _{{A1}}^{{{S_i}}}$, $\varepsilon _{{A2}}^{{{S_i}}}$ and $\varepsilon _{{A3}}^{{{S_i}}}$ are the ionospheric delay, tropospheric delay and satellite ephemeris error, respectively, of the UAV A observation satellite; $\varepsilon _{B}^{{{S_i}}}$ is the noise measurement for UAV B airborne BDS receivers; and $\varepsilon _{{B1}}^{{{S_i}}}$, $\varepsilon _{{B2}}^{{{S_i}}}$ and $\varepsilon _{{B3}}^{{{S_i}}}$ are the ionospheric delay, tropospheric delay and satellite ephemeris error, respectively, of the UAV B observation satellite.

Assuming that the UAV movement environment does not vary much, the two sub-equations in formula (8) are subtracted to eliminate the ionospheric delay, tropospheric delay, and satellite ephemeris error, and the pseudorange single difference $\Delta \rho _{{AB}}^{{{S_i}}}$ can be obtained:

9 $$\Delta \rho _{{AB}}^{{{S_i}}}=\left( {r_{A}^{{{S_i}}} - r_{B}^{{{S_i}}}} \right)+c\left( {\delta {t_A} - \delta {t_B}} \right)+\varepsilon _{{AB}}^{{{S_i}}}$$

The geodesic coordinates of the relative position vector of the two UAVs in relation to one another are defined as ${\vec {r}_{AB}}={\left( {{x_{AB}},{y_{AB}},{z_{AB}}} \right)^T}$. The position vector of satellite *S*_*i*_ with respect to the geodetic coordinate system is ${\vec {s}_i}={\left( {{x_{{S_i}}},{y_{{S_i}}},{z_{{S_i}}}} \right)^T}$. Considering two UAVs that are flying closely together, where the baseline length is short in relation to the satellite’s altitude and where the direction cosine vectors from satellite *S*_*i*_ to UAVs A and B differ only slightly^30^, we have:

10 $$\left( {r_{A}^{{{S_i}}} - r_{B}^{{{S_i}}}} \right)=\vec {\mu }_{{{S_i}}}^{T}{\vec {r}_{AB}}$$

where, ${\vec {\mu }_{{S_i}}}$ is the directional cosine vector from satellite *S*_*i*_ to the midpoint of the line connecting the two UAVs, and its expression is ${\vec {\mu }_{{S_i}}}=\frac{{{{\vec {s}}_i} - {{{{\vec {r}}_{AB}}} \mathord{\left/ {\vphantom {{{{\vec {r}}_{AB}}} 2}} \right. \kern-0pt} 2}}}{{\left\| {{{\vec {s}}_i} - {{{{\vec {r}}_{AB}}} \mathord{\left/ {\vphantom {{{{\vec {r}}_{AB}}} 2}} \right. \kern-0pt} 2}} \right\|}}$.

Substituting formula (10) into formula (9), we can obtain:

11 $$\Delta \rho _{{AB}}^{{{S_i}}}=\vec {\mu }_{{{S_i}}}^{T}{\vec {r}_{AB}}+c\left( {\delta {t_A} - \delta {t_B}} \right)+\varepsilon _{{AB}}^{{{S_i}}}$$

Similarly, the pseudo-range single difference $\Delta \rho _{{AB}}^{{{S_j}}}$ between UAVs A and B to satellite *S*_*j*_(*j* = 1,2,……,*N*, *j ≠ i*) is:

12 $$\Delta \rho _{{AB}}^{{{S_j}}}=\vec {\mu }_{{{S_j}}}^{T}{\vec {r}_{AB}}+c\left( {\delta {t_A} - \delta {t_B}} \right)+\varepsilon _{{AB}}^{{{S_j}}}$$

Formulas (11) and (12) are differentiated to eliminate the Beidou receiver clock difference and satellite clock difference to obtain the pseudo-range double difference $\Delta \left( {\Delta \rho _{{AB}}^{{{S_i}{S_j}}}} \right)$ is:

13 $$\Delta \left( {\Delta \rho _{{AB}}^{{{S_i}{S_j}}}} \right)={\left( {{{\vec {\mu }}_{{S_i}}} - {{\vec {\mu }}_{{S_j}}}} \right)^T}{\vec {r}_{AB}}+\varepsilon _{{AB}}^{{{S_i}{S_j}}}$$

Where, $\varepsilon _{{AB}}^{{{S_i}{S_j}}}$ is the noise in the pseudo-distance double difference calculation.

(2) GPS measurement model.

Assuming that both UAVs A and B are equipped with a GPS receiver, it should be installed as high as possible on the body of the UAV to prevent satellite signals from being obstructed during tilt and turning, ensuring the accuracy of the measurement output and avoiding interference from other signals. The specific installation location depends on the specifications and models of the UAV.

**Fig. 3:**
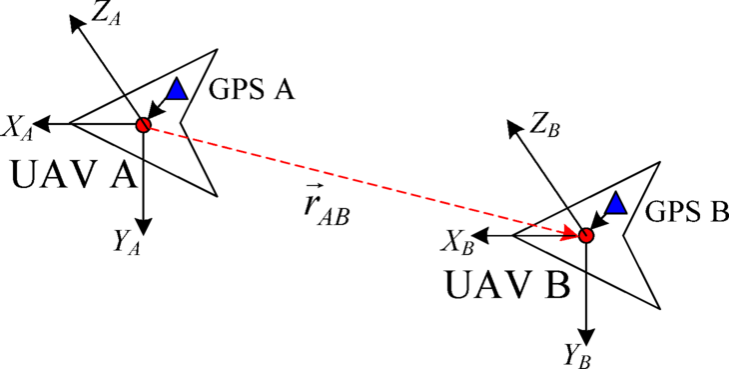
Measurement principal of the relative differential.

The relative difference method is used to establish a measurement model between the GPS measurement output and relative location in this paper, and the measurement principle is shown in Fig. 3. The installation positions of two GPS receivers relative to the body coordinate system are defined as $\left( {x_{{A0}}^{{GPS}},y_{{_{{A0}}}}^{{GPS}},z_{{_{{A0}}}}^{{GPS}}} \right)$ and $\left( {x_{{B0}}^{{GPS}},y_{{_{{B0}}}}^{{GPS}},z_{{_{{B0}}}}^{{GPS}}} \right)$. The measurement output of the GPS receiver relative to the geodetic coordinate system is (*x*^*GPS*^, *y*^*GPS*^, *z*^*GPS*^). The measurement outputs of the onboard GPS receivers of UAVs A and B relative to the geodetic coordinate system are defined as $\left( {x_{{_{A}}}^{{GPS}},y_{{_{A}}}^{{GPS}},z_{{_{A}}}^{{GPS}}} \right)$ and $\left( {x_{{_{B}}}^{{GPS}},y_{{_{B}}}^{{GPS}},z_{{_{B}}}^{{GPS}}} \right)$, respectively. Combined with the flight attitude parameters of the UAV, the position coordinates of the origin of the two drone body coordinate systems, namely the centroid coordinates, can be obtained separately:

14 $$\left\{ \begin{gathered} \left[ {\begin{array}{*{20}{c}} {{x_{OA}}} \\ {{y_{OA}}} \\ {{z_{OA}}} \end{array}} \right]={R_{{\alpha _A}}}{R_{{\beta _A}}}{R_{{\gamma _A}}}\left[ {\begin{array}{*{20}{c}} {x_{A}^{{GPS}}} \\ {y_{A}^{{GPS}}} \\ {z_{A}^{{GPS}}} \end{array}} \right] - \left[ {\begin{array}{*{20}{c}} {x_{{A0}}^{{GPS}}} \\ {y_{{A0}}^{{GPS}}} \\ {z_{{A0}}^{{GPS}}} \end{array}} \right] \hfill \\ \left[ {\begin{array}{*{20}{c}} {{x_{OB}}} \\ {{y_{OB}}} \\ {{z_{OB}}} \end{array}} \right]={R_{{\alpha _B}}}{R_{{\beta _B}}}{R_{{\gamma _B}}}\left[ {\begin{array}{*{20}{c}} {x_{B}^{{GPS}}} \\ {y_{B}^{{GPS}}} \\ {z_{B}^{{GPS}}} \end{array}} \right] - \left[ {\begin{array}{*{20}{c}} {x_{{B0}}^{{GPS}}} \\ {y_{{B0}}^{{GPS}}} \\ {z_{{B0}}^{{GPS}}} \end{array}} \right] \hfill \\ \end{gathered} \right.$$

where, $${R_{{\alpha _A}}}=\left[ {\begin{array}{*{20}{c}} {\cos {\alpha _A}}&{ - \sin {\alpha _A}}&0 \\ {\sin {\alpha _A}}&{\cos {\alpha _A}}&0 \\ 0&0&1 \end{array}} \right]$$; $${R_{{\beta _A}}}=\left[ {\begin{array}{*{20}{c}} 1&0&0 \\ 0&{\cos {\beta _A}}&{\sin {\beta _A}} \\ 0&{ - \sin {\beta _A}}&{\cos {\beta _A}} \end{array}} \right]$$; $${R_{{\gamma _A}}}=\left[ {\begin{array}{*{20}{c}} {\cos {\gamma _A}}&0&{ - \sin {\gamma _A}} \\ 0&1&0 \\ {\sin {\gamma _A}}&0&{\cos {\gamma _A}} \end{array}} \right]$$; $${R_{{\alpha _B}}}=\left[ {\begin{array}{*{20}{c}} {\cos {\alpha _B}}&{ - \sin {\alpha _B}}&0 \\ {\sin {\alpha _B}}&{\cos {\alpha _B}}&0 \\ 0&0&1 \end{array}} \right]$$; $${R_{{\beta _B}}}=\left[ {\begin{array}{*{20}{c}} 1&0&0 \\ 0&{\cos {\beta _B}}&{\sin {\beta _B}} \\ 0&{ - \sin {\beta _B}}&{\cos {\beta _B}} \end{array}} \right]$$; $${R_{{\gamma _B}}}=\left[ {\begin{array}{*{20}{c}} {\cos {\gamma _B}}&0&{ - \sin {\gamma _B}} \\ 0&1&0 \\ {\sin {\gamma _B}}&0&{\cos {\gamma _B}} \end{array}} \right]$$; (*α*_*A*_, *β*_*A*_, *γ*_*A*_) are the roll angle, pitch angle and yaw angle of UAV A, respectively, which can be obtained by extracting the on-board inertial guidance data of UAV A; and (*α*_*B*_, *β*_*B*_, *γ*_*B*_) are the roll angle, pitch angle and yaw angle of UAV B respectively, which can be obtained by extracting the on-board inertial guidance data of UAV B.

By converting the origin coordinates of body coordinate system *B* into position coordinates in body coordinate system *A*, the relative position vector $\vec {r}_{{AB}}^{{GPS}}$ between the two UAVs in body coordinate system *A* can be obtained.

15 $$\vec {r}_{{AB}}^{{GPS}}=C_{B}^{A}\left[ {\begin{array}{*{20}{c}} {{x_{OB}}} \\ {{y_{OB}}} \\ {{z_{OB}}} \end{array}} \right] - \left[ {\begin{array}{*{20}{c}} {{x_{OA}}} \\ {{y_{OA}}} \\ {{z_{OA}}} \end{array}} \right]+\vec {w}_{{AB}}^{{GPS}}=C_{B}^{A}C_{I}^{B}\left[ {\begin{array}{*{20}{c}} {x_{B}^{{GPS}}} \\ {y_{B}^{{GPS}}} \\ {z_{B}^{{GPS}}} \end{array}} \right] - C_{I}^{A}\left[ {\begin{array}{*{20}{c}} {x_{A}^{{GPS}}} \\ {y_{A}^{{GPS}}} \\ {z_{A}^{{GPS}}} \end{array}} \right] - C_{B}^{A}\left[ {\begin{array}{*{20}{c}} {x_{{B0}}^{{GPS}}} \\ {y_{{B0}}^{{GPS}}} \\ {z_{{B0}}^{{GPS}}} \end{array}} \right]+\left[ {\begin{array}{*{20}{c}} {x_{{A0}}^{{GPS}}} \\ {y_{{A0}}^{{GPS}}} \\ {z_{{A0}}^{{GPS}}} \end{array}} \right]+\vec {w}_{{AB}}^{{GPS}}$$

where, $C_{B}^{A}$ is the rotation matrix from body coordinate system *B* to body coordinate system *A*, and its expression is $C_{B}^{A}=C_{I}^{A}C_{B}^{I}=\left( {{R_{{\gamma _A}}}{R_{{\beta _A}}}{R_{{\alpha _A}}}} \right)\left( {{R_{{\alpha _B}}}{R_{{\beta _B}}}{R_{{\gamma _B}}}} \right)$; $C_{I}^{B}$ is the rotation matrix from geodesic coordinate system *I* to body coordinate system *B*, and its expression is $C_{I}^{B}={R_{{\alpha _B}}}{R_{{\beta _B}}}{R_{{\gamma _B}}}$; $C_{I}^{A}$ is the rotation matrix from geodesic coordinate system *I* to body coordinate system *B*, and its expression is $C_{I}^{A}={R_{{\alpha _A}}}{R_{{\beta _A}}}{R_{{\gamma _A}}}$; and $\vec {w}_{{AB}}^{{GPS}}$ is the noise present in the relative position calculation.

Then, through coordinate transformation, the relative position vector ${\vec {r}_{AB}}$ between the two UAVs relative to the geodetic coordinate system can be obtained as:

16 $${\vec {r}_{AB}}=C_{A}^{I}\vec {r}_{{AB}}^{{GPS}}$$

where, $C_{A}^{I}$ is the rotation matrix between body coordinate system *A* and geodetic coordinate system *I*, and its expression is $C_{A}^{I}={R_{{\gamma _A}}}{R_{{\beta _A}}}{R_{{\alpha _A}}}$.

(3) VisNav measurement model.

**Fig. 4:**
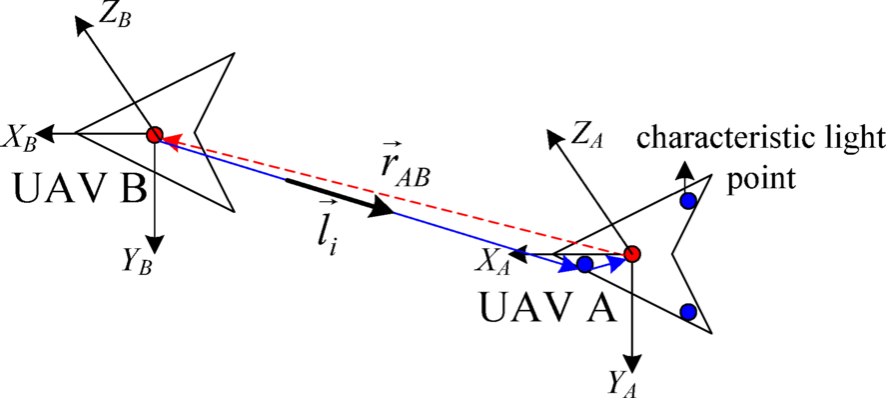
Measurement principle of the relative line of sight.

The relative line-of-sight vector method is used to determine the relative position between two UAVs. Assuming that the three characteristic light points have been marked on UAV *A*, it can be observed via the VisNav device installed on UAV *B*; the measurement principle is shown in Fig. 4^35^.

The position vector of the characteristic light point in the geodesic coordinate system is defined as ${\vec {p}_i}={\left( {{x_i},{y_i},{z_i}} \right)^T}$ (*i* = 1, 2, 3), and the relative position vector between UAVs A and B in the geodetic coordinate system is ${\vec {r}_{AB}}={\left( {{x_{AB}},{y_{AB}},{z_{AB}}} \right)^T}$. According to the measurement principle of the relative line-of-sight vector, we can obtain the unit line-of-sight vector ${\vec {l}_i}$:

17 $${\vec {l}_i}=C_{B}^{A}C_{I}^{A}{\vec {r}_i}+{\vec {\eta }_{AB}}$$

18 $${\vec {r}_i}=\frac{{\left( {{{\vec {r}}_{AB}}+{{\vec {p}}_i}} \right)}}{{\left\| {{{\vec {r}}_{AB}}+{{\vec {p}}_i}} \right\|}}=\frac{{{{\left( {{x_i}+{x_{AB}},{y_i}+{y_{AB}},{z_i}+{z_{AB}}} \right)}^T}}}{{\sqrt {{{\left( {{x_i}+{x_{AB}}} \right)}^2}+{{\left( {{y_i}+{y_{AB}}} \right)}^2}+{{\left( {{z_i}+{z_{AB}}} \right)}^2}} }}$$

Where, $C_{I}^{A}$ is the rotation matrix from the geodesic coordinate system *I* to body coordinate system *A*, and its expression is $C_{I}^{A}={R_{{\alpha _A}}}{R_{{\beta _A}}}{R_{{\gamma _A}}}$; ${\vec {\eta }_{AB}}$ is measurement noise.

### Relative positioning method of the outlier-tolerant LS

According to measurement models (12), (15), and (16), the objective function is as follows:

19 $$J\left( {{x_{AB}},{y_{AB}},{z_{AB}}} \right)=\frac{1}{2}\sum {\left( {{{\left\| {{{\left( {{{\vec {\mu }}_{{S_i}}} - {{\vec {\mu }}_{{S_j}}}} \right)}^T}{{\vec {r}}_{AB}} - \Delta \left( {\Delta \rho _{{AB}}^{{SiSj}}} \right)} \right\|}^2}} \right)} +\frac{1}{2}{\left\| {{{\vec {r}}_{AB}} - C_{A}^{I}\vec {r}_{{AB}}^{{GPS}}} \right\|^2}+\frac{1}{2}{\left\| {C_{A}^{B}C_{I}^{A}{{\vec {r}}_i} - {{\vec {l}}_i}} \right\|^2}$$

Assuming that the error vector is $\vec {\delta }={\left[ {{{\left( {{{\vec {\mu }}_{{S_i}}} - {{\vec {\mu }}_{{S_j}}}} \right)}^T}{{\vec {r}}_{AB}} - \Delta \left( {\Delta \rho _{{AB}}^{{{S_i}{S_j}}}} \right),\left( {{{\vec {r}}_{AB}} - C_{A}^{I}\vec {r}_{{AB}}^{{GPS}}} \right),\left( {C_{A}^{B}C_{I}^{A}{{\vec {r}}_i} - {{\vec {l}}_i}} \right)} \right]^T}$, formula (19) can be rewritten as:

20 $$J\left( {{x_{AB}},{y_{AB}},{z_{AB}}} \right)=\frac{1}{2}{\left\| {\vec {\delta }} \right\|^2}$$

According to the analysis in Sect. "Introduction", formula (20) does not have outlier tolerance for abnormal measurement data. To avoid the adverse effects of abnormal measurement data and ensure the reliability of the relative positioning results, combined with formula (7), formula (20) can be rewritten as follows:

21 $${J_p}\left( {{x_{AB}},{y_{AB}},{z_{AB}}} \right)=\left\{ {\begin{array}{*{20}{c}} \begin{gathered} \frac{1}{2}{\left\| {\vec {\delta }} \right\|^2}, \hfill \\ 1.5\left\| {\vec {\delta }} \right\|\left\| \sigma \right\|, \hfill \\ - {\left\| {\vec {\delta }} \right\|^2}+10\left\| {\vec {\delta }} \right\|\left\| \sigma \right\| - 18{\left\| \sigma \right\|^2}, \hfill \\ 7{\left\| \sigma \right\|^2}, \hfill \\ \end{gathered} &\begin{gathered} \left\| {\vec {\delta }} \right\| \leqslant 3\left\| \sigma \right\| \\ \\ 3\left\| \sigma \right\|<\left\| {\vec {\delta }} \right\| \leqslant 4\left\| \sigma \right\| \\ 4\left\| \sigma \right\|<\left\| {\vec {\delta }} \right\| \leqslant 5\left\| \sigma \right\| \\ \left\| {\vec {\delta }} \right\|>5\left\| \sigma \right\| \\ \end{gathered} \end{array}} \right.$$

22 $$\vec {\sigma }=\frac{1}{{m - 1}}\sum\limits_{{c=1}}^{m} {\left( {{{\vec {\delta }}_{nc}} - E\left( {{{\vec {\delta }}_{nc}}} \right)} \right){{\left( {{{\vec {\delta }}_{nc}} - E\left( {{{\vec {\delta }}_{nc}}} \right)} \right)}^T}}$$

Where, $\vec {\sigma }$ is the covariance matrix of the error vector $\vec \delta$; *m* is the sample size of the measurement data; and *n* is the sample dimension.

Correspondingly, the first derivative form of formula (21) is as follows:

23 $$\frac{{\partial {J_p}\left( {{x_{AB}},{y_{AB}},{z_{AB}}} \right)}}{{\partial \left( {{x_{AB}},{y_{AB}},{z_{AB}}} \right)}}=\left\{ {\begin{array}{*{20}{c}} \begin{gathered} \left\| {\vec {\delta }} \right\|\frac{{\partial \vec {\delta }}}{{\partial \left( {{x_{AB}},{y_{AB}},{z_{AB}}} \right)}}, \hfill \\ 1.5\frac{{\partial \vec {\delta }}}{{\partial \left( {{x_{AB}},{y_{AB}},{z_{AB}}} \right)}}\left\| \sigma \right\|, \hfill \\ - 2\left\| {\vec {\delta }} \right\|\frac{{\partial \vec {\delta }}}{{\partial \left( {{x_{AB}},{y_{AB}},{z_{AB}}} \right)}}+10\frac{{\partial \vec {\delta }}}{{\partial \left( {{x_{AB}},{y_{AB}},{z_{AB}}} \right)}}\left\| \sigma \right\|, \hfill \\ \left( {0,0,0} \right), \hfill \\ \end{gathered} &\begin{gathered} \left\| {\vec {\delta }} \right\| \leqslant 3\left\| \sigma \right\| \\ \\ 3\left\| \sigma \right\|<\left\| {\vec {\delta }} \right\| \leqslant 4\left\| \sigma \right\| \\ 4\left\| \sigma \right\|<\left\| {\vec {\delta }} \right\| \leqslant 5\left\| \sigma \right\| \\ \left\| {\vec {\delta }} \right\|>5\left\| \sigma \right\| \\ \end{gathered} \end{array}} \right.$$

Owing to the complex nonlinear function model of the measurement model, it is not possible to directly obtain the analytical solution of the function. Therefore, the Gaussian Newton iteration method^36,37^ can be used to calculate the relative positioning results.

(1) Traditional LS is used to fuse and calculate a set of initial values (*x*_*AB*0_, *y*_*AB*0_, *z*_*AB*0_);

(2) Calculate the first-order partial derivative of the objective function to obtain the corresponding Jacobian matrix ***H***;

(3) Calculation of the relative positioning results via the Gaussian-Newton iterative method.

24 $$\vec {r}_{{AB}}^{{k+1}}=\vec {r}_{{AB}}^{k} - {\left( {{H^T}H} \right)^{{\text{-}}1}}{H^T}{\vec {\zeta }^k}$$

Where, *k* is the iteration coefficient; and ${\vec {\zeta }^k}$ is the iterative error vector.

## Numerical simulation and result analysis

### Simulation environment and initialization

Computer configuration: 64-bit Windows 10 operating system; processor CPU model is Intel Xeon Silver 4215R/Tray (8c, 3.2G); graphics card model is NVIDIA GeForce Titan RTX; 32G DDR4 memory; hard disk specifications are SSD Samsung 480GB 2.5” SATA. The simulation software used was MATLAB 2019.

The BDS, GPS and VisNav are integrated to achieve the relative positioning of the two UAVs, and the inertial navigation system is used to provide the attitude angle information. The measurement accuracy of the required sensor is shown in Table 1.

**Table 1:** Sensor parameters.

Sensors	Parameters	Measured value
Gyroscope	Constant value drift	0.2(°)/h
Accelerometer	Constant value drift	100 µg
BDS	Pseudo distance error	3 m
GPS	Positioning error	5 m
VisNav	Measure noise	3500µrad

The three visible feature light points on UAV A are set, and their positions are shown in Table 2.

**Table 2:** Characteristic light spot locations.

Feature light points	X_i_/km	Y_i_/km	Z_i_/km
1	0	2.25	0
2	4.25	0	0
3	-3.75	0	2.50

The initial positions of UAVs A and B are (15, 10, 0.8) km and (10, 10, 0.8) km, respectively, and the center-of-mass motion models of UAVs A and B are as follows:

25 $$\left\{ \begin{gathered} {x_A}=15000+15000\cos \left( {{t \mathord{\left/ {\vphantom {t {600}}} \right. \kern-0pt} {600}}} \right) \hfill \\ {y_A}=10000+10000\cos \left( {{t \mathord{\left/ {\vphantom {t {600}}} \right. \kern-0pt} {600}}} \right) \hfill \\ {z_A}=800+20t \hfill \\ \end{gathered} \right.$$

26 $$\left\{ \begin{gathered} {x_B}=10000+16000\cos \left( {{t \mathord{\left/ {\vphantom {t {600}}} \right. \kern-0pt} {600}}} \right) \hfill \\ {y_B}=10000+18000\cos \left( {{t \mathord{\left/ {\vphantom {t {600}}} \right. \kern-0pt} {600}}} \right) \hfill \\ {z_B}=800+15t \hfill \\ \end{gathered} \right.$$

**Fig. 5:**
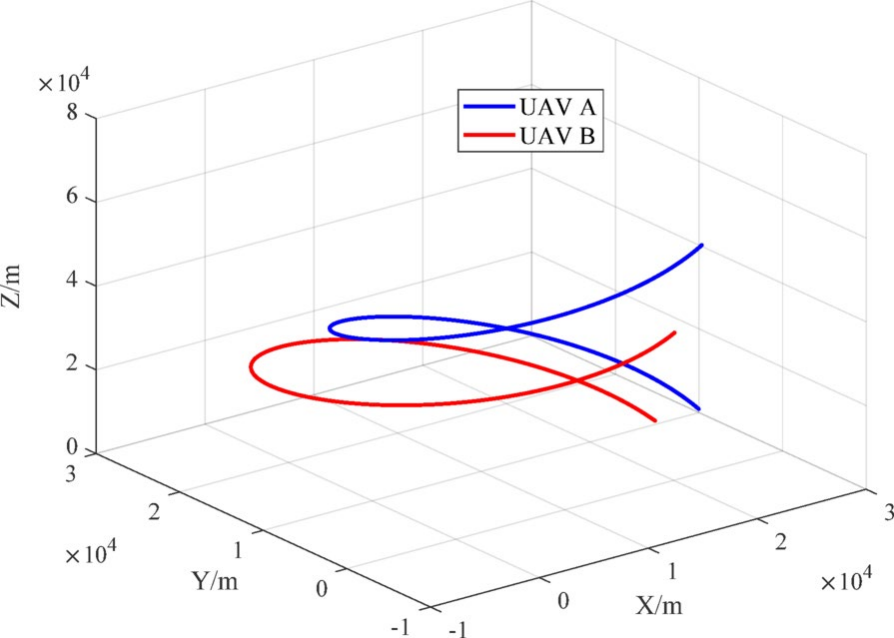
Motion trajectories of the UAVs.

**Fig. 6:**
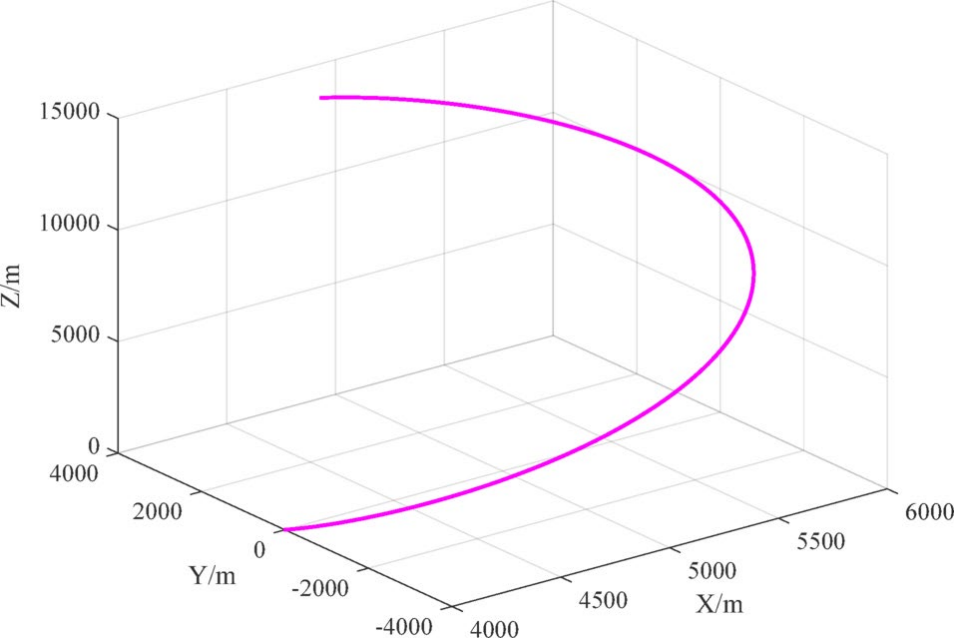
Relative positions of the UAVs.

**Fig. 7:**
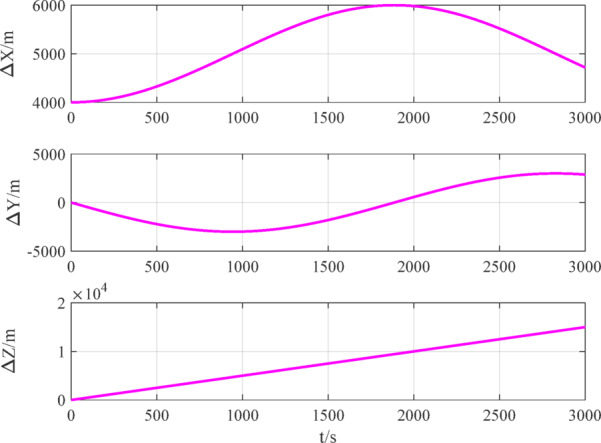
Relative positions of the UAVs.

According to motion models (25)~(26), the motion trajectories of the two UAVs are shown in Fig. 5, the relative position change of the two UAVs is shown in Fig. 6, and the three-axis component changes in the relative position are shown in Fig. 7.

**Fig. 8:**
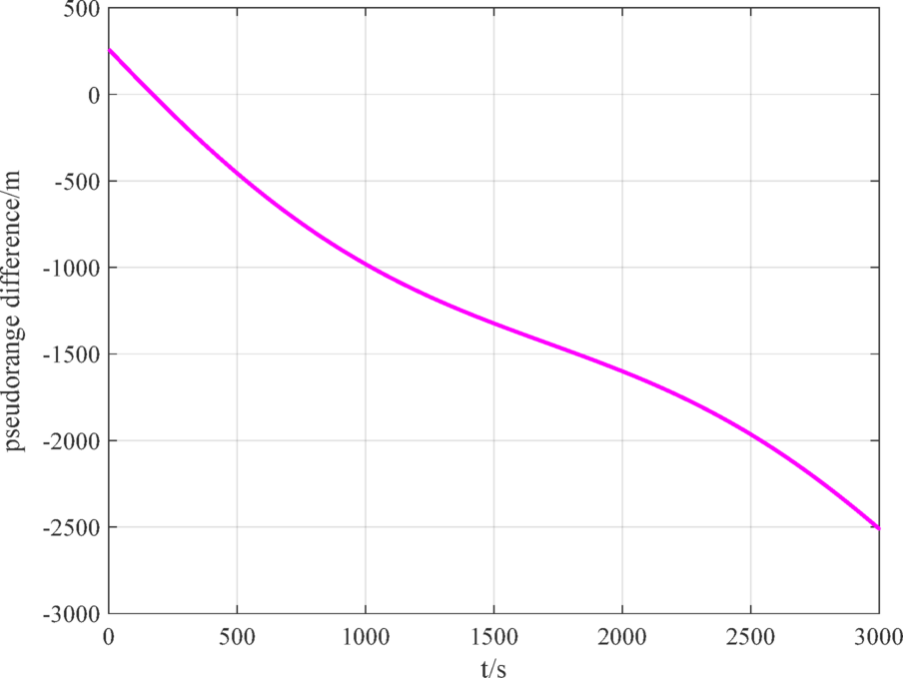
BDS measurement data.

**Fig. 9:**
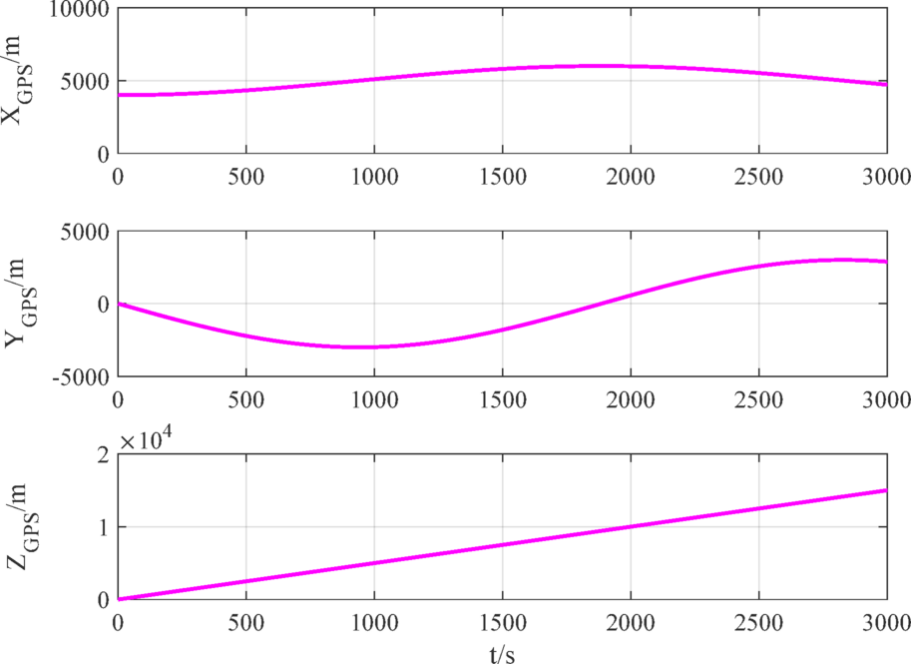
GPS measurement data.

**Fig. 10:**
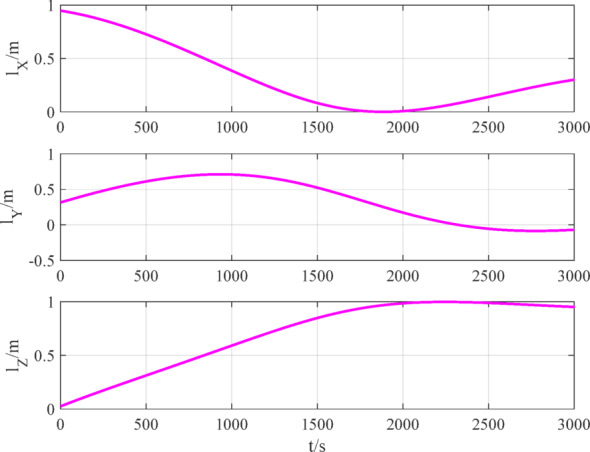
VisNav measurement data.

The number of navigation satellites used is set to 2. According to measurement models (12), (15) and (16), the measurement data of BDS, GPS and VisNav are shown in Figs. 8, 9, 10, respectively.

### Simulation results and analysis

**Fig. 11:**
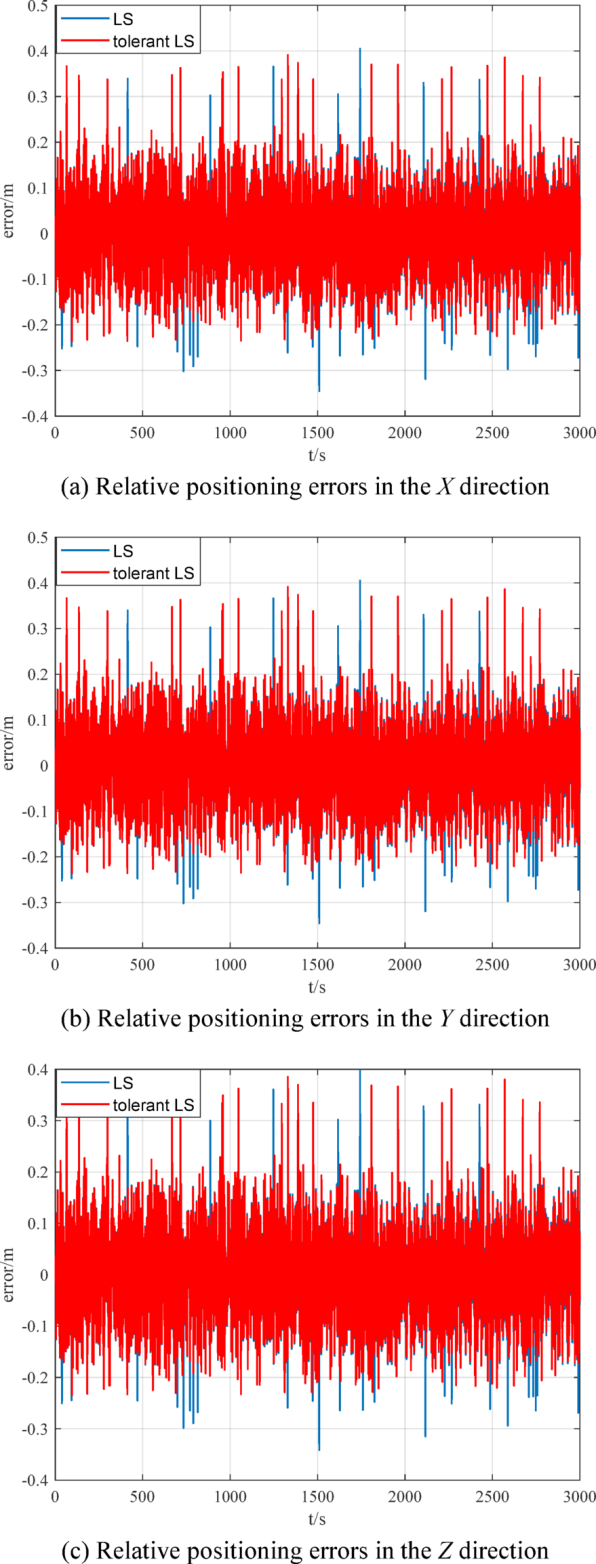
Comparison of relative positioning errors.

When there are no outliers in the measurement data of the measurement model, the relative positioning results of multi-source information fusion based on traditional LS and outlier-tolerant LS are shown in Fig. 11. The root mean square error (RMSE) of relative positioning is shown in Table 3.

**Table 3:** Comparison of relative positioning.

Relative positioning error	RMSE/m	
	traditional LS	outlier-tolerant LS
*X* direction	0.1325	0.1308
*Y* direction	0.1313	0.1305
*Z* direction	0.1301	0.1299

Figure 11; Table 3 show that in the absence of any abnormalities in the measurement data, the outlier-tolerant LS and traditional LS have similar relative positioning accuracies for multisource information fusion, with small positioning errors, high accuracy, and good reliability.

**Fig. 12:**
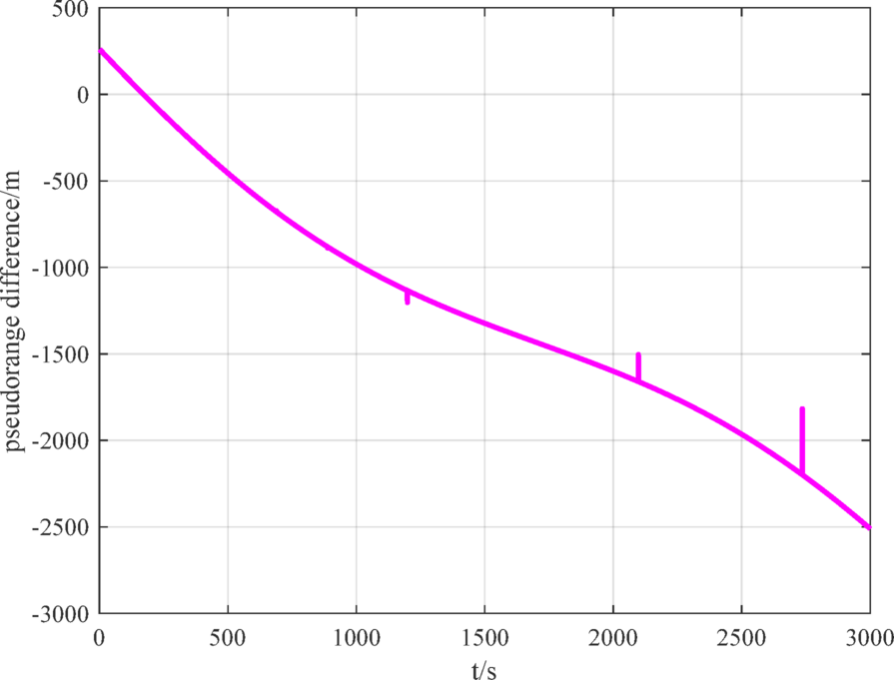
BDS measurement data with outliers.

**Fig. 13:**
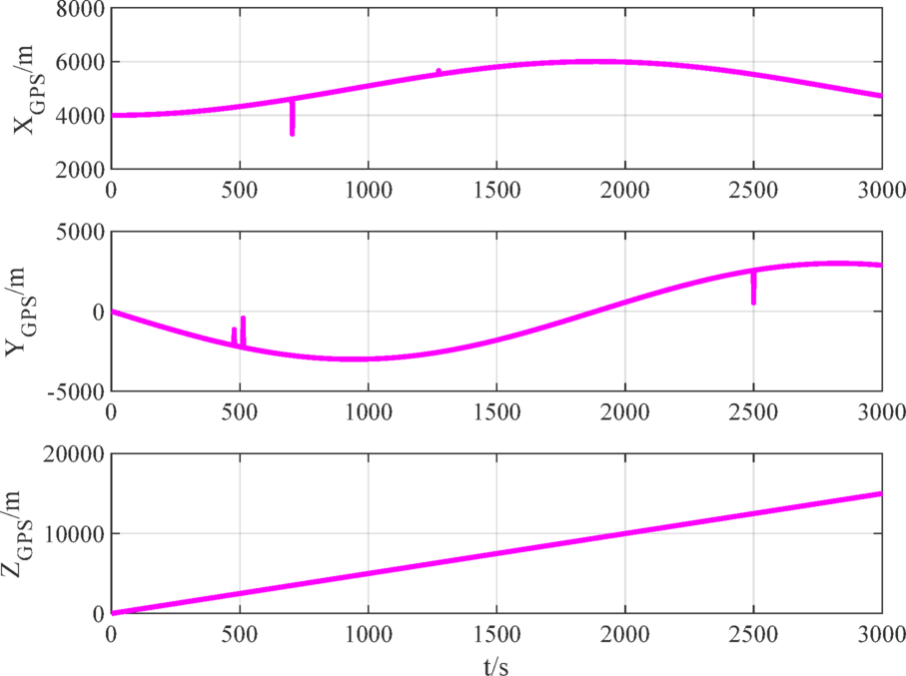
GPS measurement data with outliers.

**Fig. 14:**
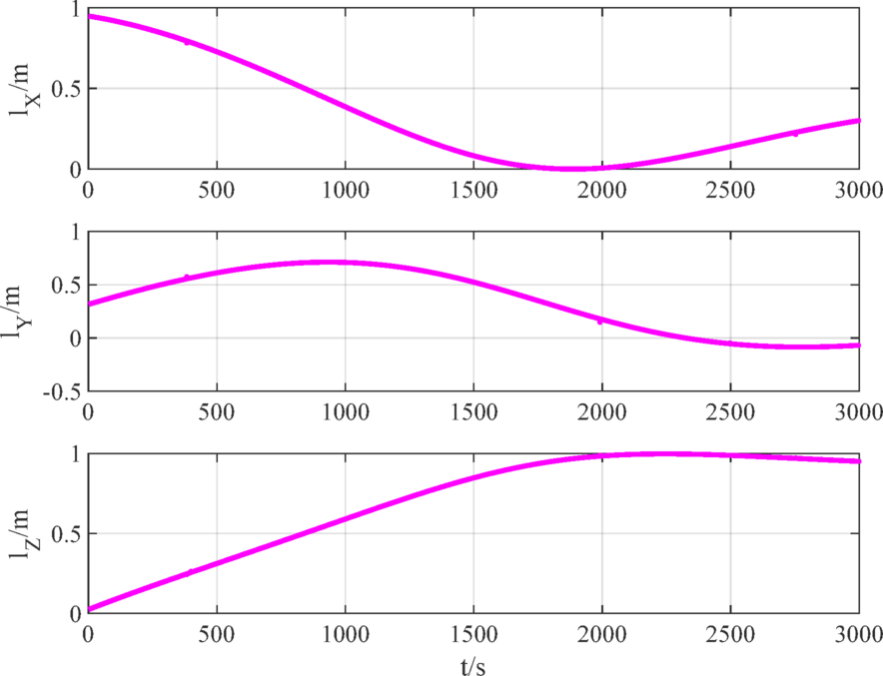
VisNav measurement data with outliers.

To verify the outlier-tolerant ability of the outlier-tolerant relative positioning method established in this paper, an external superposition method is used to set outlier point data with different biases for the measured data, as shown in Figs. 12, 13, 14.

**Fig. 15:**
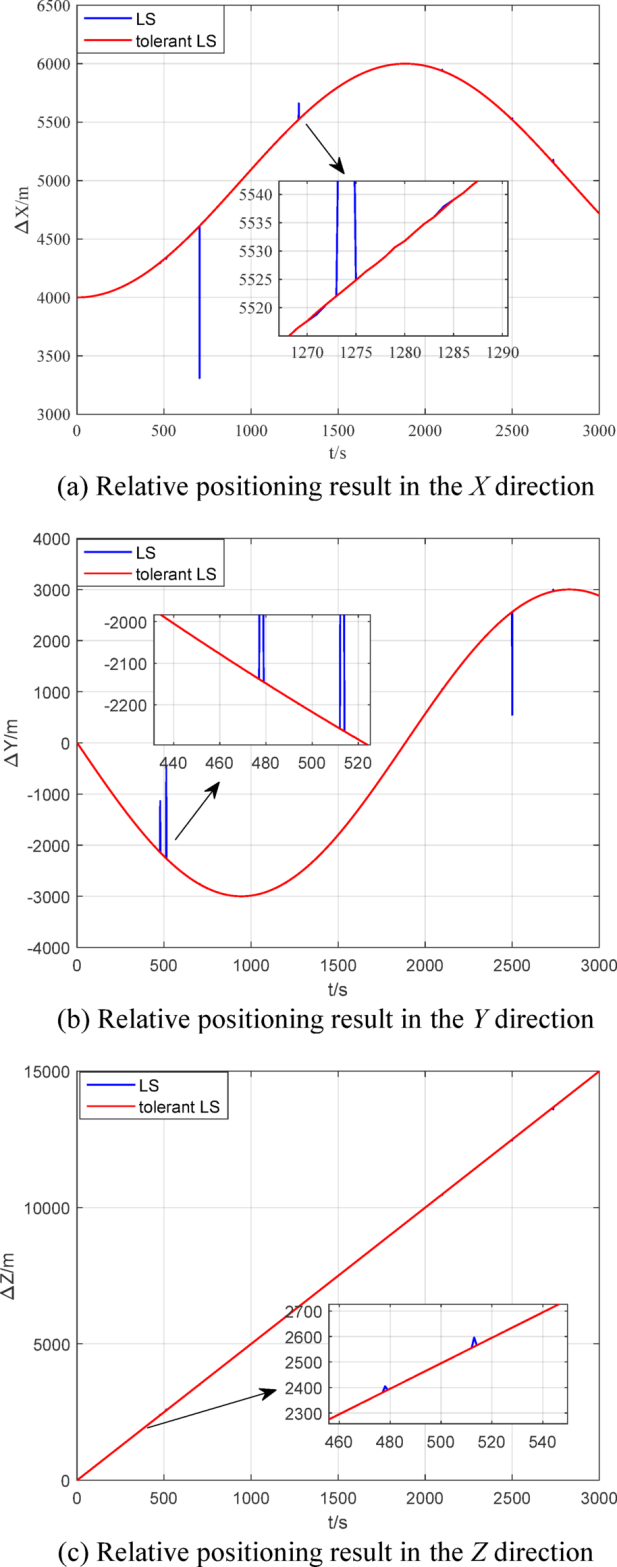
Comparison of relative positioning results.

When there are outliers in the measurement data of the measurement model, the relative positioning results of multi-source information fusion based on traditional LS and outlier-tolerant LS are shown in Figs. 15 ~ 16. The RMSE of the relative positioning is shown in Table 4.

**Fig. 16:**
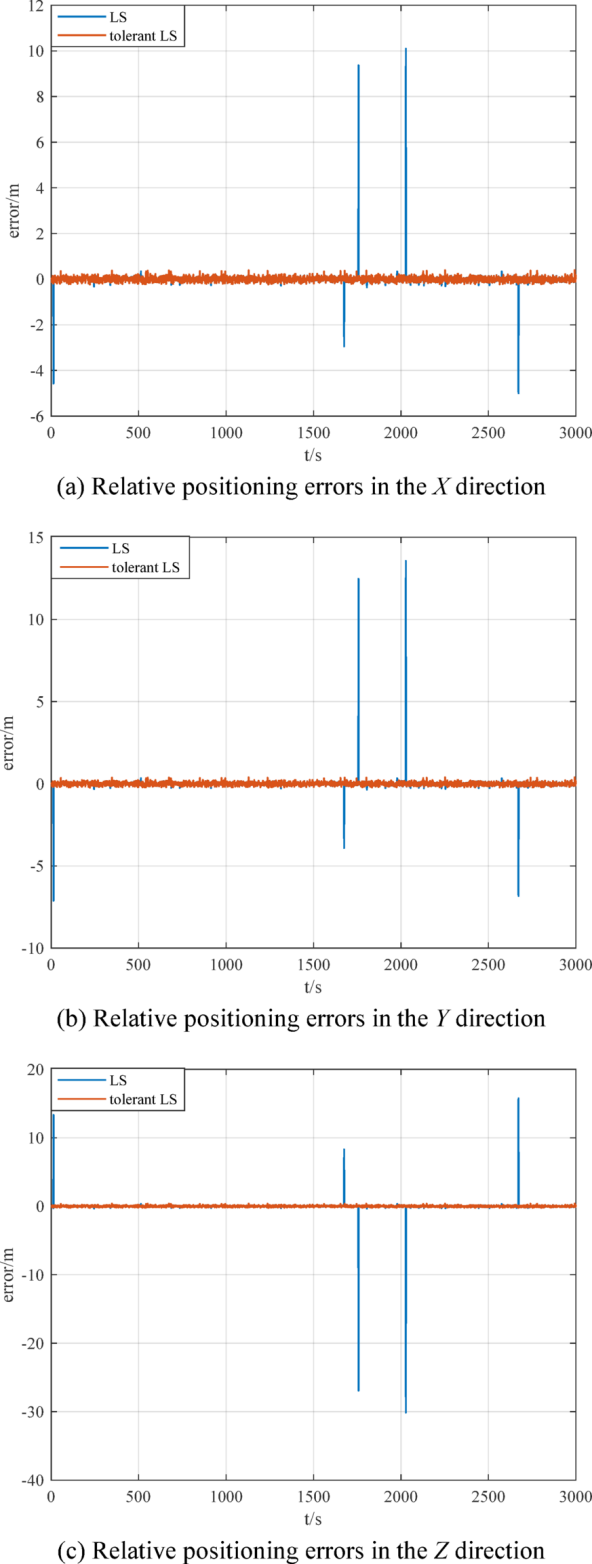
Comparison of relative positioning errors.

**Table 4:** Comparison of relative positioning.

Relative positioning error	RMSE/m	
	traditional LS	Outlier-tolerant LS
*X* direction	0.1231	0.0952
*Y* direction	0.1439	0.0953
*Z* direction	0.2441	0.0943

Figures 14 ~ 15 and Table 3 ~ 4 show that the outlier-tolerant relative positioning method based on multi-source information fusion established in this paper can not only completely eliminate the adverse effects caused by outliers in the measurement data, but also ensure the accuracy of the relative positioning result. This also indicates that the established method has good outlier tolerance ability and strong robustness and can ensure the safety and reliability of the data results.

**Fig. 17:**
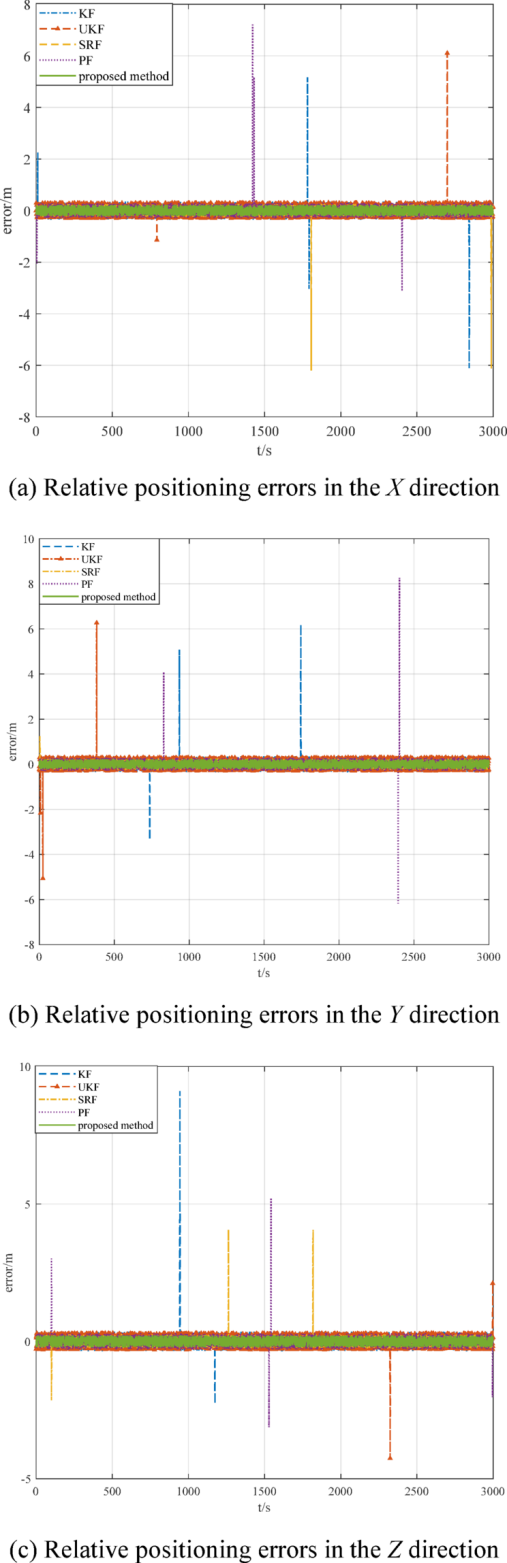
Comparison of relative positioning errors.

To further validate the performance of the proposed algorithm, a comparative analysis is conducted with typical methods such as Kalman filtering (KF), unscented Kalman filtering (UKF), square root filtering (SRF), and particle filtering (PF). The simulation results are shown in Fig. 17. The RMSE of the relative positioning is shown in Table 5.

**Table 5:** Comparison of relative positioning.

Relative positioning error	RMSE/m				
	KF	UKF	SRF	PF	Outlier-tolerant LS
*X* direction	0.1231	0.1107	0.1088	0.1011	0.0952
*Y* direction	0.1439	0.1105	0.1085	0.1015	0.0953
*Z* direction	0.2441	0.2205	0.2021	0.1875	0.0943

Figure 17; Table 5 show that the proposed method can eliminate the influence of measurement outliers and ensure the accuracy of relative navigation positioning. The comparison results demonstrate the robustness and superiority of the proposed method.

## Conclusion

In this paper, we propose an outlier-tolerant relative positioning method based on multi-source information fusion for UAVs that integrates various types of data such as satellite navigation data and visual navigation data. The proposed method can not only improve the outlier tolerance of the traditional method, but also ensure the safety and reliability of relative positioning results without rejecting abnormal data.

The results show that when the measurement data are normal, the relative positioning results of the proposed method and the traditional method are basically the same; when the measurement data are abnormal, the proposed method can avoid the adverse effects of abnormal data, ensure the reliability of the relative positioning results, and have good outlier tolerance.

The research results can achieve relative navigation positioning in complex environments, ensuring the accuracy of the navigation results in the case of abnormal navigation data. In future work, we can incorporate Galileo satellite navigation data, Glonass global satellite navigation data, and other types of data to achieve outlier-tolerant relative positioning methods with full source information fusion.

## Data Availability

The authors confirm that the data supporting the findings of this study are available within the article.

## Declarations

### Data and materials availability

The authors confirm that the data supporting the findings of this study are available within the article.
